# Integrated analysis of bulk transcriptomics and single-cell transcriptomics revealed the association characteristics between heart failure and β-hydroxybutyrylation-related genes

**DOI:** 10.1371/journal.pone.0357103

**Published:** 2026-09-18

**Authors:** Qingkuan Li, Xiyong Sheng, Guihua Li, Junjun Chen, Huayuan Zeng, Kaiyou Liu, Huating Huang, Yusheng Long, Zhihong Lu, Qingwei Ji

**Affiliations:** 1 Department of Cardiology, The People’s Hospital of Guangxi Zhuang Autonomous Region Nanning, China; 2 Guangxi University of Chinese Medicine Nanning, China; The Ohio State University, UNITED STATES OF AMERICA

## Abstract

**Background:**

As a major cause of global mortality, heart failure (HF) has mechanisms that remain elusive. Recent studies suggest β-hydroxybutyrylation (Kbhb) may contribute to its pathogenesis, driving this investigation into Kbhb-associated HF biomarkers.

**Methods:**

We collected HF-associated data and information on Kbhb-related genes (KRGs) from publicly available databases and published studies. Potential biomarker candidates were identified by determining the overlap between differentially expressed genes (DEGs) and KRGs. Subsequently, machine learning algorithms, ROC curve evaluation, and expression verification were applied to identify robust biomarkers. To investigate the biological roles of these biomarkers in HF, we performed functional enrichment analysis, assessed the immune microenvironment, predicted regulatory networks and therapeutic compounds, and conducted molecular docking simulations. Furthermore, we utilized scRNA-seq data to determine critical cell populations and examine the expression patterns of biomarkers across different cellular contexts. Additionally, we used serum samples from four heart failure patients and four healthy controls to validate the protein expression levels of the identified biomarkers using enzyme-linked immunosorbent assay (ELISA).

**Results:**

Three genes—HMOX2, H2AFZ, and KPNA2—were determined as diagnostic biomarkers for HF. Functional enrichment analyses demonstrated that these biomarkers exhibited significant associations with essential biological pathways, such as proteasome-mediated processes, oxidative phosphorylation mechanisms, and amino acid metabolic pathways. Furthermore, neutrophil infiltration showed significant positive correlations with both H2AFZ (cor = 0.42, p < 0.05) and KPNA2 (cor = 0.39, p < 0.05). Moreover, regulatory factor SP1 was predicted to target both KPNA2 and HMOX2. Thereafter, molecular docking confirmed potent binding of Bisphenol A to all 3 biomarkers, especially to HMOX2 (binding energy = −7.3 kcal/mol). Interestingly, T cells were identified as the key cell type in HF, and biomarkers exhibited dynamic changes during their differentiation. ELISA verification further confirmed that protein levels of HMOX2(P< 0.05) and KPNA2(P< 0.05) were significantly downregulated in HF patients, consistent with bioinformatics predictions. By contrast, H2AFZ exhibited a decreasing trend but without statistical significance (p> 0.05).

**Conclusion:**

HMOX2 and KPNA2 were identified as Kbhb-related biomarkers in HF, while T cells served as a key cell type in the disease. Meanwhile, this study provided novel therapeutic targets for HF patients.

## 1. Introduction

Heart failure (HF) represents a clinical syndrome resulting from structural and/or functional abnormalities of the heart, characterized by elevated rates of morbidity and mortality. This condition affects over 64 million individuals globally, with approximately 10% annual mortality, imposing substantial economic and social burdens on families and healthcare systems [1,2]. Current therapeutic strategies incorporating RAASi (renin-angiotensin-aldosterone system inhibitors), β-blockers, MRA (mineralocorticoid receptor antagonists), along with emerging therapies including ARNI (angiotensin receptor-neprilysin inhibitors) and SGLT2i (sodium-glucose co-transporter 2 inhibitors), have demonstrated effectiveness in alleviating clinical manifestations and enhancing patient outcomes. However, these treatments still do not fully reverse myocardial remodeling or completely halt disease progression, highlighting that the molecular mechanisms underlying heart failure remain only partially understood [3]. The causes of heart failure are diverse, including coronary artery disease, hypertension, diabetes among others, and heart failure itself shows significant clinical and molecular variation [4]. This suggests that current treatment strategies are not universally effective. Therefore, there is an urgent need to further explore its pathogenesis, especially by identifying key molecular pathways that drive the progression of heart failure and discovering specific biomarkers. Such advances would enable more precise diagnosis, improved risk stratification, and the development of new targeted therapies.

Emerging evidence has shown that significant metabolic reprogramming plays a crucial role in the development and progression of HF, with changes in ketone body metabolism gaining increasing attention. β-Hydroxybutyrate (BHB), one of the main circulating ketone bodies, not only acts as an alternative energy source but also functions as an epigenetic regulator by mediating lysine β-hydroxybutyrylation (Kbhb) of histone and non-histone proteins, thereby affecting gene transcription [5]. This post-translational modification was first identified by Xie et al. in mouse liver tissue [6], and later studies have confirmed its widespread presence across different tissues. Kbhb has been linked to regulating various biological processes, including oxidative stress, inflammation, and energy metabolism [7]. In diabetic cardiomyopathy models, altering Kbhb levels has been shown to significantly impact pro-inflammatory gene expression and cardiac remodeling, suggesting that Kbhb may contribute to heart disease through a metabolism-epigenetics-transcription pathway [8]. However, the expression patterns and functional roles of Kbhb-related genes in HF without diabetes are still poorly understood. Therefore, this study systematically reviewed the reported regulatory enzymes of lysine β-hydroxybutyrylation (Kbhb), including transferases and de-modifying enzymes, as well as their experimentally verified protein substrates. A total of 1492 Kbhb-related genes (KRGs) were compiled for subsequent intersection screening with differentially expressed genes in heart failure (HF) [9]. This gene set encompasses core regulatory molecules of the Kbhb modification pathway and genes encoding substrate proteins, establishing a candidate gene pool for investigating the potential roles of Kbhb in heart failure.

The current study aims to combine transcriptomic data from heart failure samples with a curated set of Kbhb-related genes (KRGs) to identify potential biomarkers strongly linked to HF. By performing differential expression analysis and using multiple algorithms for feature selection, we seek to build a functional pathway network related to Kbhb regulation. Furthermore, scRNA-seq data will be utilized to examine the expression patterns of pivotal Kbhb-related genes across distinct cardiac cell populations, revealing cell-type-specific characteristics. In summary, this investigation seeks to elucidate the functional mechanisms by which Kbhb contributes to HF pathogenesis from both molecular and cellular perspectives, thereby providing novel understanding for molecular classification and innovative therapeutic approaches in heart failure management.

## 2. Materials and methods

### 2.1. Data acquisition

Datasets related to HF were retrieved from the Gene Expression Omnibus (GEO) database (https://www.ncbi.nlm.nih.gov/geo/) on 15/04/2025, specifically GSE57338 (platform: GPL11532), GSE21610 (platform: GPL570), and the scRNA-seq dataset GSE183852 (platform: GPL24676). Among these, 82 myocardial tissue samples from patients with dilated cardiomyopathy (DCM) (HF group) and 136 control samples were selected from GSE57338. Moreover, GSE21610 contained 60 HF tissue samples (HF group) and 8 control samples (control group). Additionally, 5 HF tissue scRNA-seq samples (HF group) and 2 control samples (control group) were selected from GSE183852. Besides, a total of 1,492 KRGs were obtained from relevant literature [9] (**S1 Table**).

### 2.2. Discernment and related functional analyses of candidate genes

Genes exhibiting differential expression between HF and control cohorts were identified in GSE57338 using limma package (v 3.56.2) [10] with thresholds of Adjusted p-value (adj. p) < 0.05 and |log_2_ Fold Change (FC)| > 0.5. Visualization was achieved through volcano plots created with ggplot2 package (v 3.4.1) [11], highlighting the top 10 genes with the most pronounced up- or down-regulation based on |log_2_FC| values. The ComplexHeatmap package (v 2.14.0) [12] was employed to generate heatmaps of these highlighted genes. Candidate genes related to Kbhb in HF were identified by determining the overlap between DEGs and KRGs using the ggvenn package (v 1.7.3) [13]. Functional annotation of these candidate genes was conducted through GO and KEGG pathway enrichment analyses (p < 0.05) via the clusterProfiler package (v 4.2.2) [14], referencing the org.Hs.e.g.,db package (v 3.16.0) [15]. To clarify the directionality of pathway enrichment, the upregulated and downregulated candidate genes were analyzed separately. Results were ranked by ascending p-values, selecting the top 5 terms from each GO category (BPs, CCs, MFs) and all KEGG pathways for visualization. Protein interaction networks were constructed using the STRING database (https://www.string-db.org) with a confidence threshold of 0.4 (accessed on 19/04/2025), and interactive components were visualized through Cytoscape software (v 3.9.1) [16] after excluding isolated nodes.

### 2.3. Determination of biomarkers

Three distinct machine learning approaches were applied to GSE57338 for screening Kbhb-associated biomarkers from candidate genes. LASSO regression analysis was performed using the glmnet package (v 4.1.4) [17]with 10-fold cross-validation (family = binomial), identifying genes with non-zero coefficients at lambda.min as LASSO features. Concurrently, SVM-RFE analysis via the e1071 package (v 1.7–13) [18] selected features based on minimal error and maximal accuracy criteria through 10-fold cross-validation. Additionally, the Boruta package (v8.0.0) [19] identified characteristic features using default settings through shadow feature integration and iterative comparisons. Key genes were defined as the intersection of features from all three methods using the ggvenn package (v 1.7.3). Discriminative performance was assessed using the pROC package (v 1.18.0) [20] to generate ROC curves and calculate AUC values in both GSE57338 and GSE21610 datasets, with AUC > 0.7 indicating satisfactory discrimination. Candidate biomarkers meeting this criterion were further validated by comparing expression patterns between HF and control groups via Wilcoxon test (p < 0.05) in both datasets, with consistently differentially expressed genes designated as final biomarkers.

### 2.4. Development and evaluation of nomogram

A predictive nomogram for HF risk assessment was constructed in GSE57338 using the regplot package (v 1.1) [21], assigning individual scores to each biomarker with cumulative scores indicating elevated HF risk. Clinical utility was evaluated through DCA using the rmda package (v 1.6) [22]. Model performance was validated by generating ROC curves with the pROC package (v 1.18.0) and calculating AUC values (acceptable performance: AUC > 0.7).

### 2.5. Gene set enrichment analysis (GSEA)

To explore biomarker-associated biological pathways in HF progression, GSEA was performed using the “c2.cp.kegg_legacy.v2024.1.Hs.symbols.gmt” gene set from MSigDB (https://www.gsea-msigdb.org/gsea/msigdb) (accessed on 20/04/2025). Spearman correlation coefficients between each biomarker and all genes in GSE57338 were calculated using the psych package (v 2.2.9) [23], with genes ranked by correlation strength. GSEA was conducted via the clusterProfiler package (v 4.2.2) with criteria of |NES| > 1 and p.adjust < 0.05, visualizing the top 5 pathways ranked by p.adjust values.

### 2.6. Immune microenvironment analysis

Immune cell infiltration in GSE57338 was quantified using the CIBERSORT algorithm within the IOBR package [24] to estimate abundances of 22 immune cell types [25], visualized through heatmaps. Wilcoxon test identified significantly different immune cell populations between groups (p < 0.05), designated as differentially infiltrated cells. Correlations between biomarkers and these immune cells were evaluated using Spearman analysis via the psych package (v 2.2.9) with thresholds of p < 0.05 and |cor| > 0.3.

### 2.7. Construction of regulatory network

TFs and miRNAs regulating biomarkers were predicted using the Networkanalyst database (https://www.networkanalyst.ca/) (accessed on 22/04/2025), with regulatory networks visualized through Cytoscape software (v 3.9.1) to illustrate TF-biomarker-miRNA interactions.

### 2.8. Drug prediction and molecular docking

Potential therapeutic agents targeting biomarkers were identified using DSigDB (http://tanlab.ucdenver.edu/dsigdb/), with the top 10 drugs per biomarker (ranked by binding scores) visualized via Cytoscape software (v 3.9.1). Molecular docking was performed for the highest-scoring drug per biomarker, obtaining protein structures from PDB (http://www.rcsb.org) (accessed on 20/04/2025) and drug structures from PubChem (https://pubchem.ncbi.nlm.nih.gov/) (accessed on 20/04/2025). Docking simulations were conducted using CB-Dock2 (https://cadd.labshare.cn/cb-dock/php/blinddock.php) on (accessed on 20/04/2025), with binding energies below 0 indicating spontaneous binding and values ≤ −5.0 kcal/mol suggesting stable complex formation.

### 2.9. Identification of the key cell type through scRNA-seq analysis

To investigate the molecular mechanisms underlying heart failure (HF) development, we performed comprehensive scRNA-seq analysis using the GSE183852 dataset, with particular focus on identifying critical cell populations in HF pathogenesis. The Seurat package (v 5.0.1) [26] was employed for batch effect removal from the scRNA-seq data. We implemented stringent quality control measures to ensure cell quality: (1) cells expressing between 200 and 4,000 genes (nFeature_RNA) were selected; (2) cells showing expression counts (nCount_RNA) up to 10,000 were included, while genes detected in a minimum of 3 cells were preserved; (3) cells with mitochondrial gene content (percent.mt) below 10% were retained. Data normalization was achieved through the NormalizeData function. We identified 2,000 highly variable genes (HVGs) via the FindVariableFeatures function, and the LabelPoints function enabled visualization and annotation of the 10 most variable genes. Data scaling was accomplished using the ScaleData function. Using these 2,000 HVGs as input, we conducted principal component analysis (PCA) on the processed dataset through the RunPCA function. The JackStraw function facilitated selection of statistically significant principal components (PCs) by identifying those enriched for genes with p-values below 0.05. Concurrently, we generated an elbow plot using the ElbowPlot function to determine the appropriate PC number for dimensional reduction.

We performed unsupervised clustering with a resolution parameter of 0.4 using the FindNeighbors and FindClusters functions to define cell clusters. Uniform Manifold Approximation and Projection (UMAP) enabled dimensional reduction and unbiased two-dimensional visualization of cell populations. The RunUMAP function generated UMAP visualizations for cluster representation. Cell cluster identities were assigned based on established marker genes from published literature [27]. Dot plots illustrated marker gene expression patterns across clusters. Clusters were annotated based on genes showing unique or elevated expression patterns, with each cluster assigned to its corresponding cell type. We subsequently assessed cell type abundance differences between HF patients and controls. Statistical comparison of biomarker expression across cell types between groups was performed using the Wilcoxon rank-sum test (p < 0.05). Through integrated analysis incorporating existing literature, we identified the pivotal cell type contributing to HF progression.

### 2.10. Cellular communication and pseudo-temporal trajectory analyses

The CellChat package (v 1.6.1) [28]was utilized to analyze the ligand-receptor (LR) interaction pairs among annotated cells from GSE183852, and construct a network, thereby comprehensively revealing interactions among different annotated cell types. In order to comprehensively analyze the heterogeneity of key cells, the key cell type was subjected to batch effect removal, dimensionality reduction (based on PCs with p < 0.05), clustering (resolution = 0.3), and annotation again to observe the different subtypes of the key cell type. The selection of marker genes was performed with reference to relevant literature [29,30]. The monocle package (v 2.26.0) [31] was utilized to predict the differentiation states of the key cell type, while the DDRTree function from the same package was employed to map their differentiation trajectories. Thereafter, the monocle package (v 2.26.0) was also employed to delineate the dynamic trend of biomarkers expression across the process of key cell differentiation.

### 2.11. Patients with HF and Healthy Controls

We obtained blood samples from 4 patients with heart failure and 4 healthy controls from the Congestive Heart Failure Registry of Diagnosis and Therapy in Guangxi (GREAT) (registration number: ChiCTR2100041989). This study was approved by the Medical Ethics Committee of the People’s Hospital of Guangxi Zhuang Autonomous Region (approval number: KY-DZX-202006).

All clinical samples were fully anonymized before acquisition and analysis. No prospective participant recruitment was performed for the present study, and all samples were collected under the ethical framework of the original GREAT registry study, in which all participants provided written informed consent prior to inclusion. The clinical samples used for research purposes in this study were obtained on 01/08/2025.

### 2.12. Sample Processing and ELISA-Based Protein Quantification

To obtain serum samples, blood specimens were allowed to stand at room temperature for 1 hour, followed by centrifugation at 4,000 rpm for 15 minutes at 4°C using a refrigerated centrifuge.To validate the protein expression levels of candidate genes identified via bioinformatics analysis, enzyme-linked immunosorbent assay (ELISA) was performed following the manufacturer’s protocol: (1) Equilibrated all reagents to room temperature, diluted the test samples, and prepared serial gradient dilutions of the standard; (2) Added 50 μL of samples or standards to the pre-coated microplate wells, respectively, and incubated at 37°C for 30 minutes; (3) Washed the plate 5 times, then added HRP-labeled enzyme conjugate and incubated continuously at 37°C for 30 minutes; (4) Washed the plate another 5 times, added chromogenic solutions A and B, and incubated at 37°C in the dark for 10 minutes; (5) Added stop solution to terminate the reaction, and measured the optical density (OD) at 450 nm using a microplate reader within 15 minutes; (6) Calculated the target protein concentrations in the samples based on the standard curve (fitted by linear regression). All ELISA kits used in this experiment were purchased from Jiangsu Meimian Industrial Co., Ltd.

### 2.13. Statistical analysis

Statistical analyses were conducted using R software (v 4.1.0), or GraphPad Prism 10(Version 10.1.2) Between-group differential comparisons were performed through the Wilcoxon rank-sum test, with statistical significance defined as p < 0.05. The significance levels in all figures were denoted as follows: **** indicated p < 0.0001, *** indicated p < 0.001, ** indicated p < 0.01, * indicated p < 0.05, while ns (not significant) indicated p > 0.05.

## 3. Results

### 3.1. Discovery of 15 candidate genes and investigation into their biological roles

**Fig 1:**
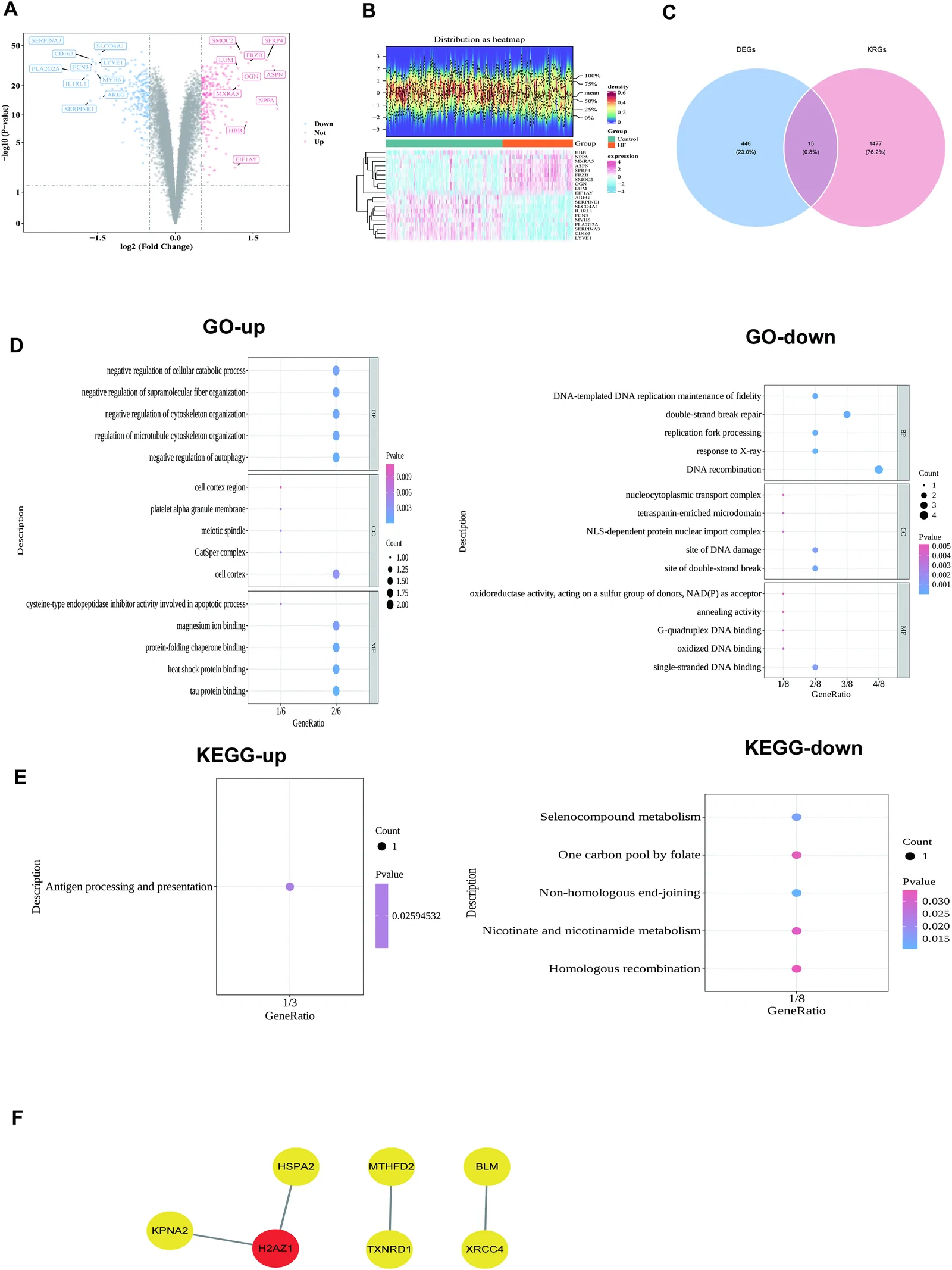
Discovery of 15 candidate genes and investigation into their biological roles. (**A-B**) The volcano plot and heatmap of differentially expressed genes (DEGs) between the heart failure (HF) and control groups in GSE57338; (**C**) Venn of DEGs and β-hydroxybutyrylation (Kbhb)-related genes (KRGs); (**D-E**) Gene Ontology (GO) and Kyoto Encyclopedia of Genes and Genomes (KEGG) analyses of candidate genes; (**F**) Protein-protein interaction (PPI) network of candidate genes.

After performing differential analysis on the GSE57338 to distinguish DEGs between HF and control groups, a total of 461 DEGs were obtained. Specifically, when compared to the control group, 223 genes were down-regulated, and 238 genes were up-regulated in the HF group. Results were presented using a volcano plot and a heatmap (Fig 1A and 1B). Taking the intersection of 461 DEGs and 1,492 KRGs yielded 15 candidate genes (Fig 1C), among which 6 were upregulated (SNCA, RPS4Y1, HSPA2, ZNF536, PHLDB2, and STK38L) and 9 were downregulated (BLM, SAMHD1, HMOX2, NAMPT, XRCC4, MTHFD2, TXNRD1, KPNA2, and H2AFZ). Moreover, the upregulated candidate genes significantly accumulated within 336 GO terms (p < 0.05), comprising 288 biological processes (BPs), 24 cellular components (CCs), and 24 molecular functions (MFs), whereas the downregulated candidate genes significantly accumulated within 165 GO terms (p < 0.05), comprising 122 BPs, 13 CCs, and 30 MFs. (Fig 1D, **S2 Table**). For the upregulated candidate genes, the top 5 enriched BPs included “negative regulation of cellular catabolic process” and “negative regulation of supramolecular fiber organization.” The top 5 enriched CCs included “cell cortex region” and “platelet alpha granule membrane.” The top 5 enriched MFs included “cysteine-type endopeptidase inhibitor activity involved in apoptotic process” and “magnesium ion binding.” For the downregulated candidate genes, the top 5 enriched BPs included “DNA-templated DNA replication maintenance of fidelity” and “double-strand break repair.” The top 5 enriched CCs included “nucleocytoplasmic transport complex” and “site of DNA damage.” The top 5 enriched MFs included “oxidoreductase activity, acting on a sulfur group of donors, NAD(P) as acceptor” and “annealing activity.” In addition, KEGG analysis showed that the upregulated candidate genes were enriched in only 1 pathway (p < 0.05): “Antigen processing and presentation.” The downregulated candidate genes were enriched in 5 pathways (p < 0.05): “Selenocompound metabolism,” “One carbon pool by folate,” “Non-homologous end-joining,” “Nicotinate and nicotinamide metabolism,” and “Homologous recombination.” (Fig 1E). The PPI network showed that H2AZ1 could interact with KPNA2 and HSPA2, while MTHFD2 interacted with TXNRD1, and BLM interacted with XRCC4 (Fig 1F). These results provided preliminary evidence for the association between HF and Kbhb.

### 3.2. Acquisition of 3 biomarkers: HMOX2, H2AFZ, and KPNA2

**Fig 2:**
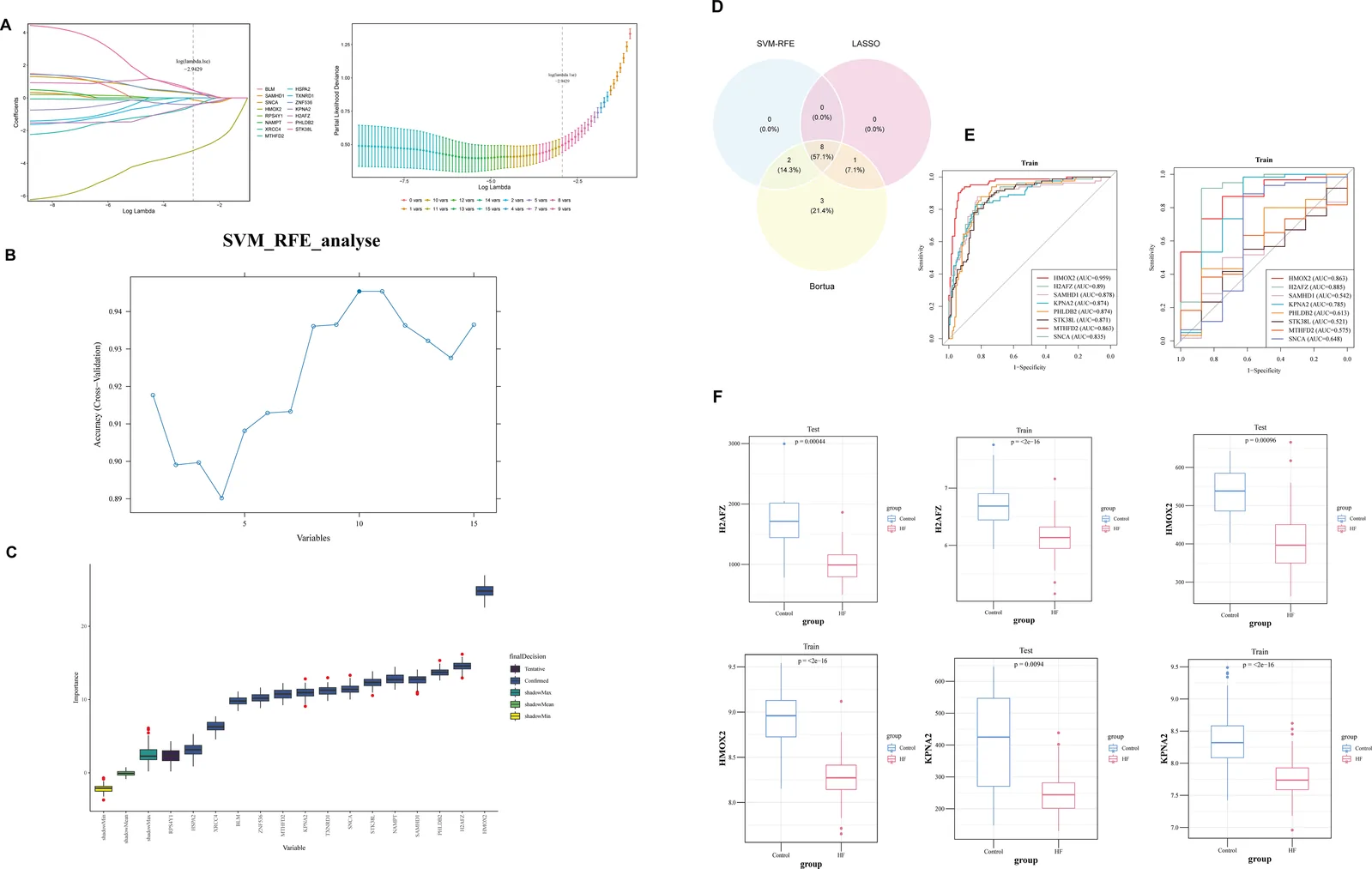
HMOX2, H2AFZ, and KPNA2 were identified as biomarkers for HF. (**A**) The path plot of regression coefficients and cross-validation curve for the least absolute shrinkage and selection operator (LASSO) algorithm; (**B**) The support vector machine-recursive feature elimination (SVM-RFE) method; (**C**) The Boruta algorithm; (**D**) Venn diagram of the results from 3 algorithms; (**E**) The receiver operating characteristic (ROC) curves of key genes in GSE57338 and GSE21610; (**F**) The box plots of the expression levels of candidate biomarkers in GSE57338 and GSE21610.

In the LASSO regression algorithm, when the log(lambda.min) was −2.9429, the model was optimal, and 9 characteristic genes were obtained, namely SAMHD1, SNCA, HMOX2, MTHFD2, ZNF536, KPNA2, H2AFZ, PHLDB2, and STK38L (Fig 2A). Moreover, when the number of selected features was set to 10, the SVM-RFE model attained its maximum level of accuracy. The 10 genes that were selected at this stage were HMOX2, H2AFZ, NAMPT, SAMHD1, KPNA2, PHLDB2, STK38L, MTHFD2, BLM, and SNCA (Fig 2B). Meanwhile, Boruta algorithm screened 14 characteristic genes (Fig 2C). The genes procured by the 3 algorithms were subsequently intersected to identify 8 key genes, namely HMOX2, H2AFZ, SAMHD1, KPNA2, PHLDB2, STK38L, MTHFD2, and SNCA (Fig 2D). Subsequently, it was found that HMOX2, H2AFZ, and KPNA2 all exhibited a good ability to distinguish between HF samples and control samples in GSE57338 and GSE21610 (AUC > 0.7) (Fig 2E). Additionally, their expression levels in the HF group were significantly lower than those in the control group (Fig 2F). Therefore, these 3 genes were selected as Kbhb-related biomarkers in HF for subsequent analyses.

### 3.3. The nomogram performed favourably in diagnosing HF

**Fig 3:**
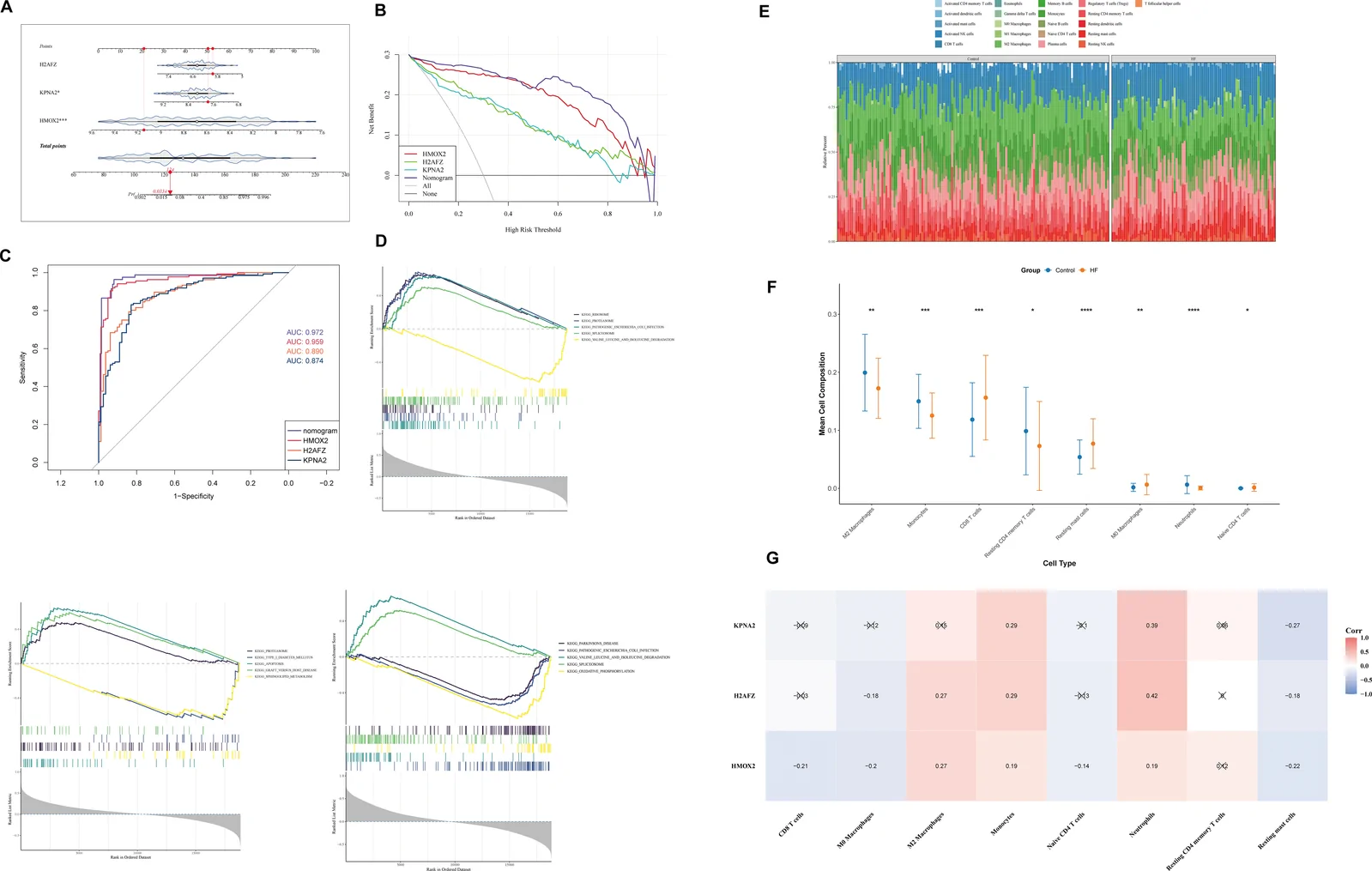
Development of the nomogram and functional investigation of HMOX2, H2AFZ, and KPNA2. (**A**) Nomogram constructed based on 3biomarkers; (**B-C**) The decision curve analysis (DCA) curves (**B**) and ROC curves (**C**) of the nomogram; (**D**) Gene set enrichment analysis (GSEA) of HMOX2, H2AFZ, and KPNA2; (**E**) Heatmaps of 22 immune cell infiltration levels in GSE57338; (**F**) Dot plot of infiltration levels of 8 differentially infiltrated immune cells between the control and HF groups; (**G**) Correlation analysis of biomarkers and differentially infiltrated immune cells.

To further assess the diagnostic utility of HMOX2, H2AFZ, and KPNA2, a predictive nomogram was developed. The nomogram assigned points to the 3 biomarkers and calculated total points to predict the probability of HF occurrence. The nomogram revealed that a total score of 124 corresponded to a 0.0334 probability of HF occurrence, and elevated total points from HMOX2, H2AFZ, and KPNA2 were associated with an increased probability of HF (Fig 3A). The DCA curves confirmed that the nomogram had great clinical utility (Fig 3B). The ROC curve revealed that the nomogram showed really high diagnostic value (AUC = 0.972) (Fig 3C). To sum up, a well-constructed nomogram was developed, and the calibration and ROC curves demonstrated its favorable predictive performance.

### 3.4. The biological functions of HMOX2, H2AFZ, and KPNA2 were further explored

Further exploration was conducted on the biological functions of the biomarkers. GSEA revealed that H2AFZ was significantly enriched in 68 pathways, HMOX2 in 31 pathways, and KPNA2 in 62 pathways (p.adjust < 0.05, |NES| > 1) (**S3 Table**). Notably, both H2AFZ and HMOX2 showed significant enrichment in the “proteasome” pathway. In addition, H2AFZ and KPNA2 were co-enriched in “pathogenic escherichia coli infection” and “valine, leucine and isoleucine degradation.” Furthermore, KPNA2 was specifically enriched in the “oxidative phosphorylation” pathway (Fig 3D). The above results facilitated in-depth understanding of the biological functions of the 3 biomarkers.

### 3.5. Significant relationships between immune infiltrating cells and biomarkers

The immune microenvironment between the HF and control groups was subjected to further analysis. Fig 3E visualized the immune infiltration profiles of 22 immune cell types in GSE57338. The infiltration levels of 8 immune cell types exhibited significant disparities between the HF and control groups (p < 0.05). Among them, CD8 T cells and others showed higher infiltration in the HF group, whereas M2 macrophages and other subsets were more abundant in the control group (Fig 3F). In addition, both H2AFZ (cor = 0.42, p < 0.05) and KPNA2 (cor = 0.39, p < 0.05) were significantly positively correlated with neutrophil infiltration (Fig 3G). These findings would facilitate a more profound examination of the immune microenvironment in HF, thereby establishing the foundation for comprehending its pathogenesis and formulating targeted intervention strategies.

### 3.6. Exploration of the regulatory network and molecular docking for HMOX2, H2AFZ, and KPNA2

**Fig 4:**
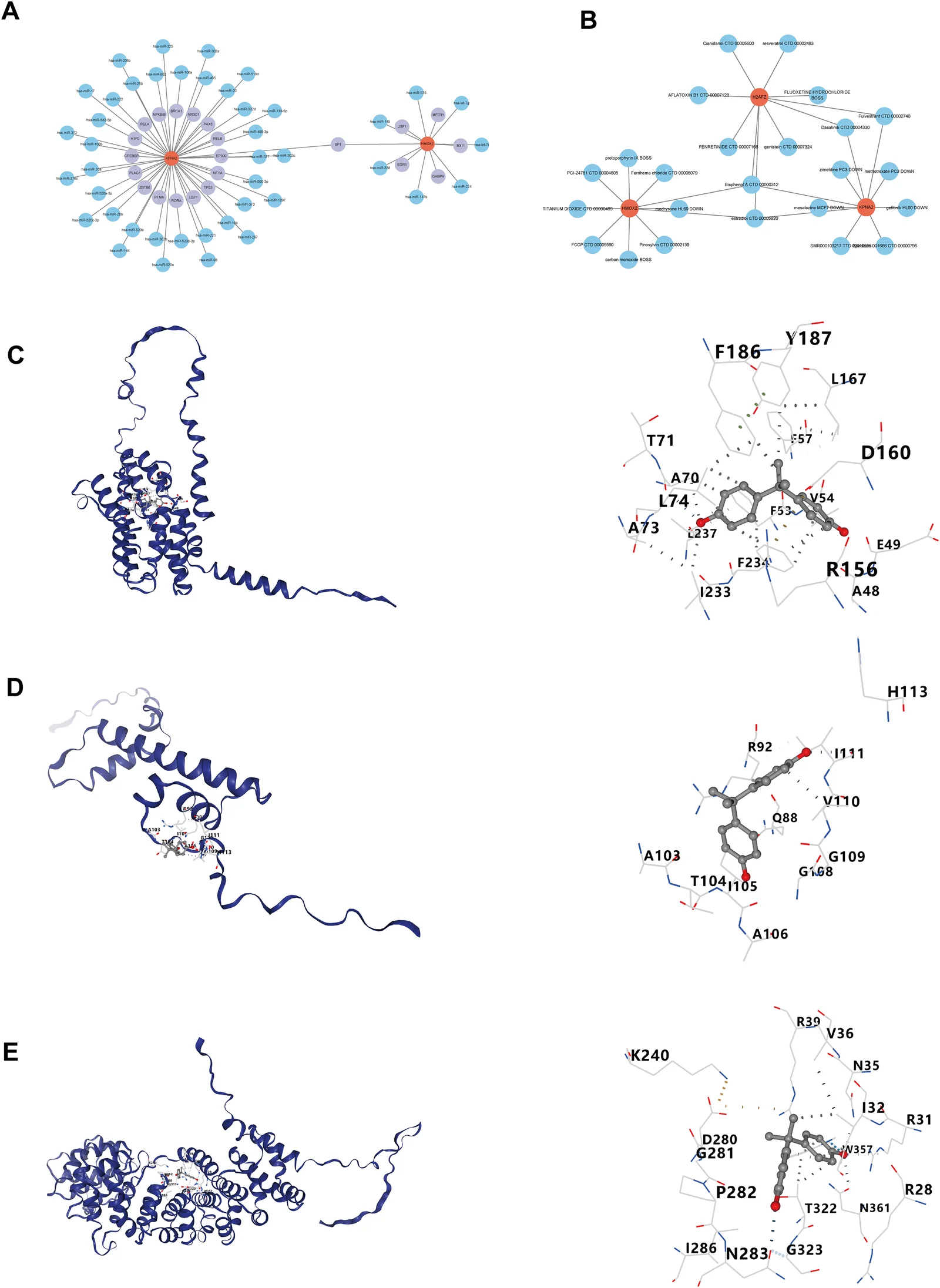
Exploration of the regulatory network and molecular docking for HMOX2, H2AFZ, and KPNA2. (**A**) The microRNAs (miRNAs)-biomarkers-transcription factors (TFs) network, red: biomarkers, blue: miRNAs, grey: TFs; (**B**) The biomarkers-drugs network, red: biomarkers, blue: drugs; (**C-E**) The molecular docking results of Bisphenol A and HMOX2 (**C**), H2AFZ (**D**), and KPNA2 (**E**).

As illustrated in Fig 4A, bioinformatic prediction identified 36 miRNAs and 17 TFs targeting KPNA2, along with 7 miRNAs and 6 TFs regulating HMOX2. Notably, SP1 was predicted to target both KPNA2 and HMOX2, whereas no regulatory factors were identified for H2AFZ. Furthermore, drug targeting the identified biomarkers were predicted via the DSigDB database. Specifically, 40, 16, and 51 potential drugs were identified for HMOX2, H2AFZ, and KPNA2, respectively (**S4 Table**). A network diagram displayed the top 10 drugs ranked by binding score for each biomarker, revealing that Bisphenol A and estradiol could simultaneously target all 3 biomarkers (Fig 4B). Based on their high binding scores across all 3 targets, Bisphenol A was selected for molecular docking. The results indicated spontaneous binding between Bisphenol A and each biomarker, with the strongest binding affinity observed for HMOX2 (binding energy = −7.3 kcal/mol) (**Table 1**). Detailed interaction analysis showed that Bisphenol A formed contacts with residues D160 and R156 in HMOX2 (Fig 4C), potentially interacted with I105 in H2AFZ (Fig 4D), and formed hydrogen bonds with W357 in KPNA2 (Fig 4E). These results not only clarify the potential regulatory network underlying HF but also provide a candidate drug (Bisphenol A) and its precise binding sites for subsequent in vitro/in vivo validation of HF-targeted therapies.

**Table 1:** Binding energy of molecular docking between each biomarkers (HMOX2, H2AFZ, and KPNA2) and Bisphenol A.

Genes	Drugs	Binding energy(kcal/mol)
H2AFZ	Bisphenol A	−5.3
KPNA2	Bisphenol A	−6.8
HMOX2	Bisphenol A	−7.3

### 3.7. T cells were identified as the key cell type

**Fig 5:**
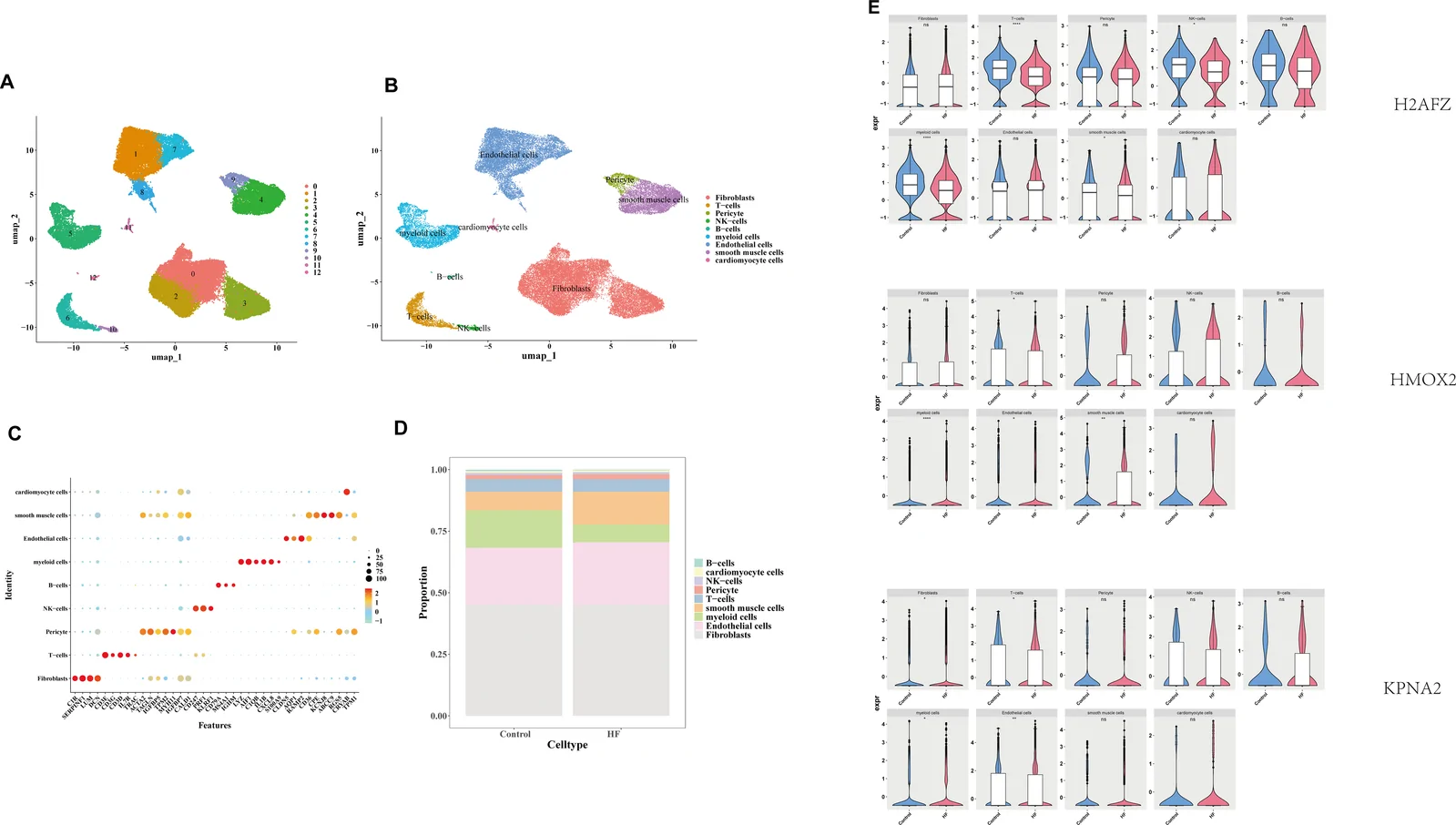
T cells were identified as the key cell type. (**A**) UMAP plot of 12 cell clusters; (**B**) Cell annotation by UMAP; (**C**) Expression of marker genes in different annotated cells; (**D**) Distribution proportions of different annotated cells; (**E**) The expression differences of biomarkers in different annotated cells between the control and HF groups.

Following the implementation of QC procedures for the GSE183852, the cell count was reduced from 49,042–45,300, while the gene count remained constant at 23,963 (**S1****A and** **S1B Fig**). Next, 2,000 HVGs were identified, and the top 10 HVGs were annotated (**S1C Fig**). Further, PCA was performed, and the top 20 PCs were selected for further analysis (p < 0.05) (**S1****D and** **S1E Fig**). Thereafter, the 13 clusters were visualized utilizing the UMAP method (resolution = 0.2) (Fig 5A). Employing marker genes facilitated the identification of 9 distinct cell types: fibroblasts, endothelial cells, T cells, pericyte, smooth muscle cells, cardiomyocytes, natural killer (NK) cells, myeloid cells, B cells (Fig 5B). The specific marker genes associated with each distinct annotated cell type were detailed in Fig 5C and **Table 2**. Furthermore, both the HF and control groups were predominantly composed of endothelial cells and fibroblasts. Compared to the control, the HF group exhibited higher proportions of endothelial cells and smooth muscle cells, whereas a greater abundance of myeloid cells was observed in the control group (Fig 5D). Further analysis revealed significant differences in the expression levels of HMOX2, H2AFZ, and KPNA2 within T cells and myeloid cells between the 2 groups (Fig 5E). Based on these findings and supported by prior literature [32], T cells were selected as the key cellular context for subsequent analysis in HF.

**Table 2:** Marker genes for cell type annotation.

Cell_name	Marker_gene
Fibroblasts	C1R, SERPINF1, LUM, DCN
endothelial cells	CLDN5, AQP1, RAMP2
T-cells	CD3E, CD3G, CD3D, IL7R, TRAC
Pericyte	ACTA2, TAGLN, IGFBP5, TPM2, MYH11, IGFBP7, CALD1
smooth muscle cells	CD36, CPE, KCNJ8, ABCC9, RGS5
cardiomyocytes	CRYAB, TPM1
NK-cells	CD247, PRF1, KLRF1
myeloid cells	LYZ, AIF1, C1QB, IL1B, CXCL8, S100A9
B-cells	CD79A, MS4A1, IGHM

### 3.8. Comprehensive analysis of the pathological mechanisms of HF at the cellular level

**Fig 6:**
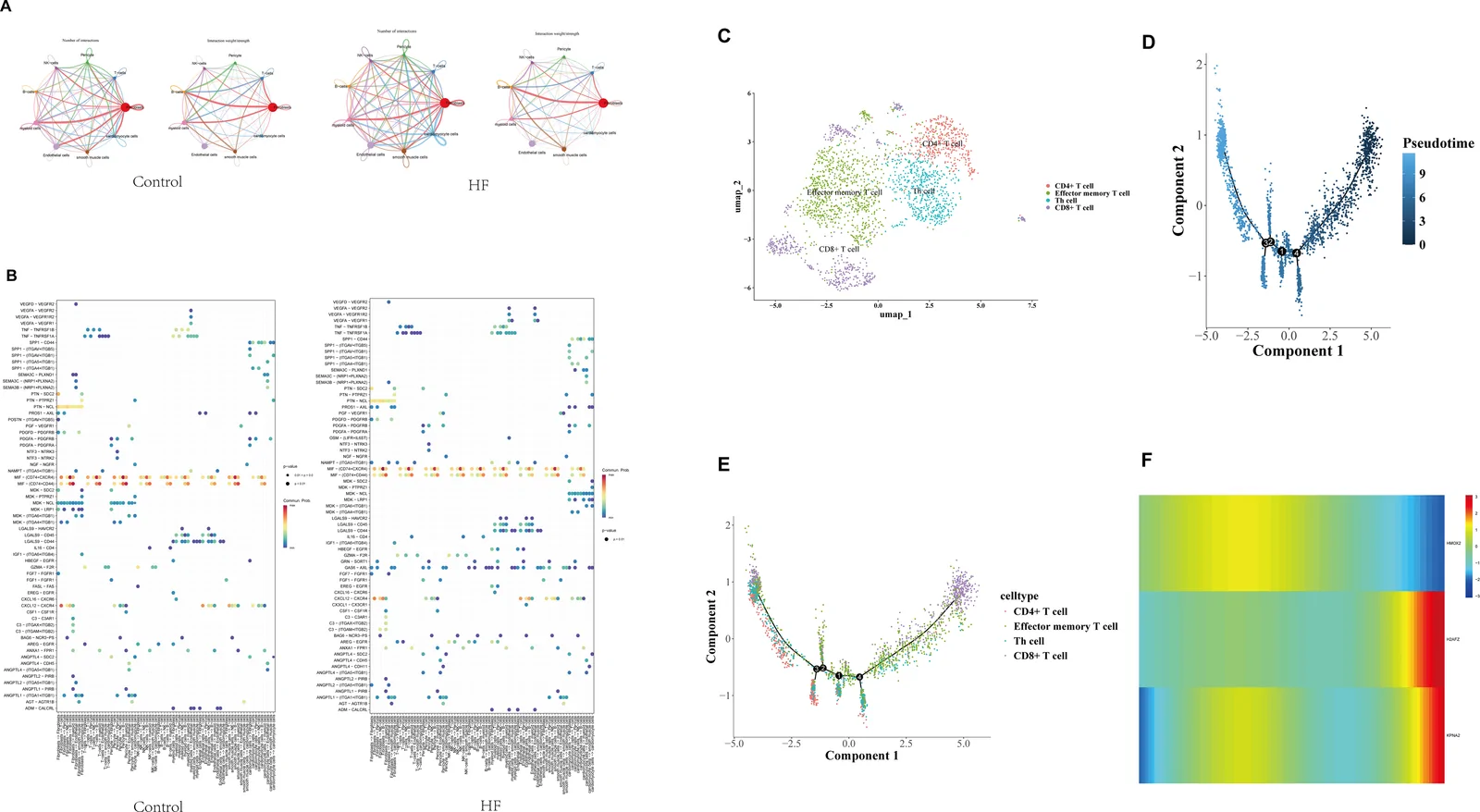
Cellular communication and pseudo-temporal trajectory analyses. (A-B) Cellular communication of different annotated cells; (C) Cell annotation for subsets of T cells visualized on UMAP plot; (D-E) Pseudo-temporal trajectory analysis of T cells; (F) Expression changes of biomarkers observed during T cell differentiation.

Cell communication analysis revealed a complex interaction network among the nine annotated cell types. The results indicated that fibroblasts exhibited extensive communication with other cell types in both the HF and control groups. Furthermore, the interaction strength between T cells and other populations, including NK cells and cardiomyocytes, was higher in the HF group than in the control (Fig 6A). Notably, in the HF group, T cell interactions were predominantly mediated by the MIF-(CD74 + CXCR4) and MIF-(CD74 + CD44) LR interaction pairs. A similar pattern was observed in the control group, where the same pairs also facilitated the majority of T cell communications (Fig 6B). To further explore the roles of T cells in HF, separate clustering analyses of this cell type population were conducted. The results showed that T cells were divided into 9 clusters (resolution = 0.3) (**S2****A and** **S2C Fig**). Subsequently, 4 subsets were identified using marker genes, namely CD4^+^ T cells, CD8^+^ T cells, helper T cells (Th cells), and effector memory T cells (Fig 6C, **S2D Fig****, and Table 3**). Following this, the comprehensive description of the pseudo-temporal trajectory analysis pertaining to T cells was provided. The differentiation process of T cells over pseudo-time was illustrated in Fig 6D and 6E. During the differentiation process, CD8 ⁺ T cells were detected almost exclusively in the early and late stages, while CD4 ⁺ T cells emerged predominantly during the mid-to-late phase. In contrast, Th cells and effector memory T cells were distributed throughout nearly the entire differentiation timeline. In terms of gene expression, HMOX2 showed low expression in the early phase of T cell differentiation but was nearly absent by the terminal stage. Conversely, H2AFZ and KPNA2 were primarily expressed in the late differentiation phase (Fig 6F). Based on comprehensive single-cell profiling, this study delineated the dynamic distributions of T cell subsets and stage-specific expression patterns of biomarkers throughout the differentiation of T cells, providing crucial insights into the cellular basis of HF.

**Table 3:** Marker genes for T cell subset annotation.

Cell_name	Marker_gene
CD4 + T cells	LEF1, NOSIP
Effector memory T cells	CD27, GZMK
Th cells	CD28, ICOS
CD8 + T cells	CCL5, CD8A, CD8B

### 3.9. Experimental Validation of Candidate Protein Expression in HF Patients and Controls

**Fig 7:**
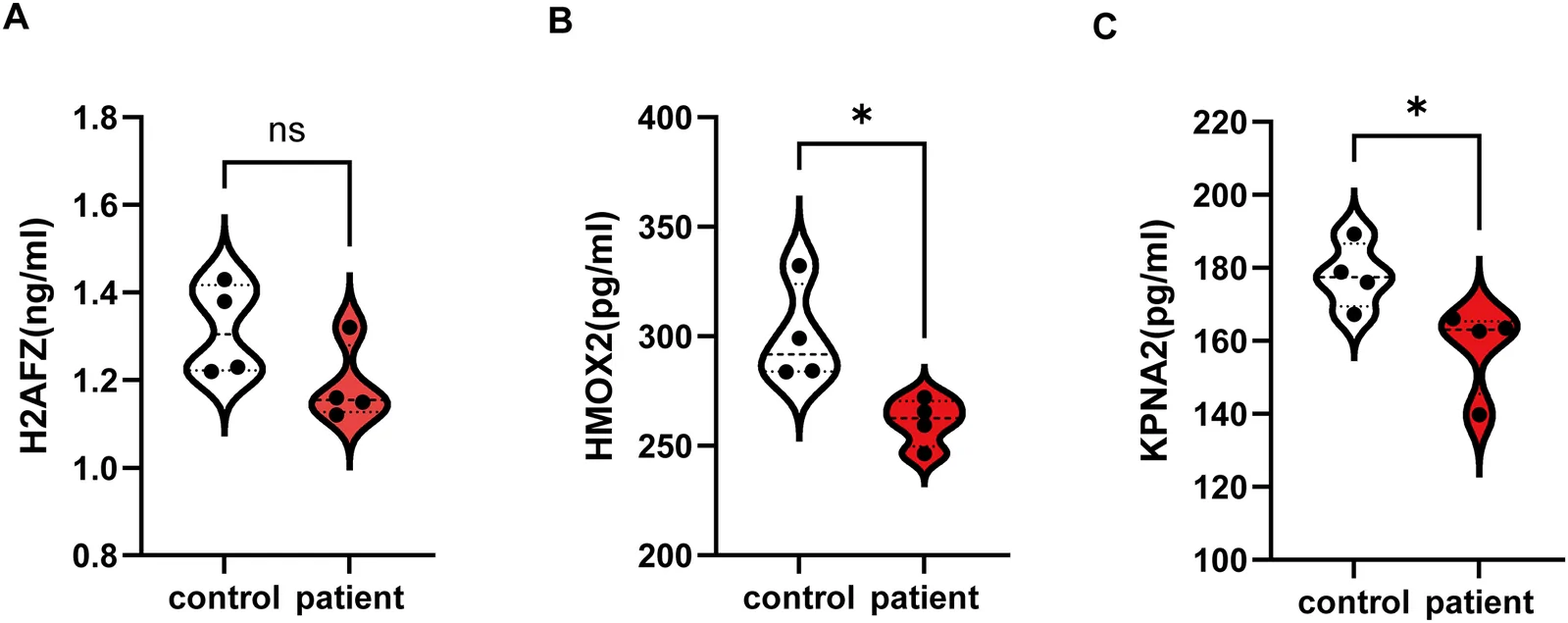
ELISA validation of candidate protein expression levels in HF patients and healthy controls. (**A**)H2AFZ protein level in the HF group showed a decreasing trend compared with the control group, with no statistical significance; (**B**)HMOX2 protein expression in the HF group was significantly lower than that in the control group. (**C**)KPNA2 protein expression in the HF group was significantly lower than that in the control group.

Compared with the control group, the protein level of H2AFZ exhibited a decreasing trend in HF patient group, with no statistically significant difference observed (Fig 7A). The expression level of HMOX2 protein in HF patients was significantly lower than that in the control group (Fig 7B), which was consistent with the expression trend predicted by bioinformatics analysis. Additionally, the protein level of KPNA2 in the HF patient group was significantly downregulated compared with the control group (Fig 7C), which also matched the results obtained from bioinformatics screening.

## 4. Discussion

HF severely impairs patients’ quality of life and imposes a heavy socioeconomic burden. Abnormal modification of Kbhb can cause metabolic reprogramming in the myocardium, contributing to the progression of heart failure. In this study, we integrated bioinformatics analysis with clinical sample validation to explore the relationship between HF and Kbhb modifications. We obtained transcriptomic data related to HF and KRGs from public databases. Through comprehensive transcriptomic analysis, we identified three Kbhb-related biomarkers(HMOX2, H2AFZ and KPNA2), and conducted an in-depth analysis of their roles in HF. Moreover, Enrichment analysis revealed that these genes are significantly involved in various essential biological pathways, particularly those related to cellular physiological activities. Additionally, single-cell RNA sequencing analysis identified T cells as key players in HF and unveiled their specific pathological characteristics. This research provides a scientific basis for elucidating the pathophysiological mechanisms underlying heart failure.

As a key enzyme that catalyzes the breakdown of heme into carbon monoxide and bilirubin, heme oxygenase 2 (HMOX2) plays a vital role in maintaining vascular relaxation, reducing oxidative stress, and preserving cellular homeostasis [33]. We demonstrated in our experiments that the serum HMOX2 protein levels in patients with heart failure were significantly lower than those in healthy controls, which was fully consistent with the downregulation trend of HMOX2 at the transcriptome level in the GSE57338 and GSE21610 datasets. Its decreased expression may result in lower production of antioxidant metabolites, weakening the ability of cardiomyocytes to combat reactive oxygen species (ROS)-induced damage. Additionally, our analysis showed that HMOX2 was enriched in pathways related to the proteasome, apoptosis, and sphingolipid metabolism, indicating that its dysregulation could worsen myocardial injury and functional decline through effects on cell death pathways and lipid balance. These findings align with previous studies demonstrating that HMOX2 deficiency in mice leads to impaired cardiac development and contractility, ultimately causing cardiomyopathy [34].

The H2A family member Z (H2AFZ) is a crucial histone variant regulating chromatin architecture, thereby influencing DNA replication, repair, and transcription [35,36]. Its dysregulation is implicated in various diseases. While elevated H2AFZ can promote oncogenesis [37,38], its decrease may compromise genomic stability [39,40]. Its specific role in HF remains unclear. In our study, H2AFZ was significantly downregulated in HF models. Enrichment analysis linked it to ribosome, proteasome, spliceosome, and branched-chain amino acid degradation pathways. Its downregulation may disrupt chromatin dynamics, leading to transcriptional insufficiency in these pathways, potentially causing imbalances in protein homeostasis, RNA splicing, and metabolism (e.g., valine degradation). Notably, our ELISA validation showed serum H2AFZ protein levels had a decreasing trend in HF patients but without statistical significance. This may be attributed to two factors: first, the limited sample size (n = 4 per group) may have underpowered the detection of subtle differences; second, as a nuclear histone, H2AFZ is poorly reflected in serum. Direct assessment in myocardial tissue via IHC or Western blot is warranted in future studies.

Karyopherin Subunit Alpha 2 (KPNA2) is a key nuclear import protein that facilitates the transport of transcription factors and cell cycle regulators, governing gene expression, proliferation, and differentiation. Its dysregulation is linked to multiple diseases [41–47]. In our study, KPNA2 protein expression was significantly downregulated in HF patients, consistent with transcriptomic data from failing myocardium. This downregulation may impair the nuclear import of pro-angiogenic factors like STAT3, compromising compensatory angiogenesis and exacerbating energy deficit [48]. Furthermore, reduced KPNA2—potentially via enhanced CHIP-mediated degradation—could alleviate its inhibition on the NF-κB pathway, amplifying myocardial inflammation [49]. Notably, we found KPNA2 associated with the oxidative phosphorylation pathway; its deficiency may hinder nuclear import of related regulators, contributing to the ATP synthesis crisis in HF. Together with Mendelian randomization evidence linking KPNA2 to atherosclerosis [50], these mechanisms form a multidimensional network through which KPNA2 influences HF progression. Collectively, KPNA2, along with HMOX2 and H2AFZ, promotes cardiomyocyte dysfunction, death, and a malignant inflammatory cycle, suggesting that multi-target interventions against this network represent a promising therapeutic direction.

Our enrichment analysis revealed significant enrichment of the biomarkers in the oxidative phosphorylation pathway, highlighting its crucial role in the development and progression of HF. Oxidative phosphorylation is the primary physiological process in mitochondria responsible for ATP production. As a tissue with high energy demands, the heart’s normal pumping function relies heavily on the integrity of mitochondrial oxidative metabolism [51,52]. Studies have shown that dysfunction of oxidative phosphorylation in HF, such as decreased efficiency and reduced complex activity, directly leads to insufficient ATP production in cardiomyocytes, resulting in weakened myocardial contractility. Additionally, disturbances in oxidative phosphorylation are often accompanied by excessive ROS generation, which further aggravates oxidative damage and mitochondrial dysfunction in cardiomyocytes. In this study, we observed that KPNA2 was specifically enriched in the “oxidative phosphorylation” pathway, indicating it may impair ATP synthesis by affecting the nuclear transport of regulatory proteins involved in the electron transport chain (ETC) complexes. This mechanism could contribute to an inadequate energy supply in cardiomyocytes, worsening myocardial dysfunction.

Immune infiltration analysis revealed a significant increase in the proportion of CD8 + T cells and a decrease in the proportion of M2 macrophages in the myocardium of the HF group. These changes in the immune cell subpopulation composition suggest remodeling of the myocardial immune microenvironment in HF. At the metabolic-epigenetic regulatory level, it has been shown that the metabolic product BHB, through Kbhb modification, can enhance the PHD2-HIF1-α signaling pathway in macrophages, promoting VEGF production and angiogenesis [53]. Additionally, the loss of HMOX2 weakens its antioxidant function, exacerbating tissue oxidative stress, which in turn activates CD8 + T cells and inhibits the polarization of M2 macrophages, the latter of which have repair functions [54]. Meanwhile, the downregulation of H2AFZ may disrupt the transcriptional programs of genes required for maintaining immune homeostasis, potentially mediated by epigenetic mechanisms such as Kbhb-dependent modification or others. The reduction in KPNA2 may impair the nuclear transport of inflammation-related transcription factors, such as NF-κB, further amplifying the inflammatory signal. Therefore, the dysfunction of these Kbhb-related genes collectively form a pro-inflammatory [55], anti-repair metabolic-epigenetic state, thereby directly driving myocardial immune imbalance characterized by T cell infiltration and the loss of M2 macrophages, ultimately accelerating the progression of heart failure.

In molecular docking analysis, we found that bisphenol A (BPA) exhibited strong binding activity with the three biomarkers, HMOX2, H2AFZ, and KPNA2. BPA is a common endocrine disruptor with a structure similar to estrogen. It is widely used in medical devices, food packaging, and plastic products, and can easily be absorbed by the human body through ingestion or skin contact [56]. Previous studies have shown that BPA levels in blood or urine are positively correlated with various cardiovascular metabolic diseases and may induce myocardial dysfunction through multiple signaling pathways [57–60]. Based on our results, we hypothesize that BPA might promote HF progression through three main mechanisms: disrupting KPNA2-mediated nuclear transport and oxidative phosphorylation, inhibiting the antioxidant activity of HMOX2, and interfering with chromatin dynamics regulated by H2AFZ. Furthermore, these mechanisms may act together synergistically. For example, HMOX2 inhibition can lead to ROS accumulation, KPNA2 dysfunction may promote the nuclear translocation of inflammatory factors, and H2AFZ abnormalities could impair myocardial contractile function. Collectively, these alterations might establish a “oxidative–inflammation–structural disorder” feedback loop, accelerating HF onset and progression. These findings suggest that BPA could be a significant risk factor for HF. Future research could focus on developing targeted antagonists based on its binding patterns with the three biomarkers or on reversing its damaging effects via regulation of related gene expression, opening new avenues for HF treatment strategies.

Analysis of single-cell sequencing data revealed that HMOX2, H2AFZ, and KPNA2 exhibit cell-type-specific expression patterns across different myocardial cell subpopulations. In single-cell trajectory analysis, it was observed that HMOX2 expression was mainly concentrated in the early stages of T cell differentiation, especially in the CD4 + T cell and naïve Th cell clusters, where its levels were higher. This indicates that HMOX2 may have an antioxidant regulatory role during the initial activation and functional maintenance of T cells. Conversely, H2AFZ and KPNA2 showed higher expression in later-stage effector and memory T cell clusters, particularly in effector memory T cells and CD8 + T cells.Therefore, the three genes are collectively proposed as HF biomarkers based on comprehensive consideration of their capacity to capture pathological T cell characteristics at distinct differentiation stages. Rather than requiring them to exhibit consistent expression trends, this combination enables a more comprehensive depiction of the immune-metabolic-epigenetic crosstalk network underlying heart failure (HF). In addition, it should be noted that the CIBERSORT analysis was performed using bulk RNA-seq data from GSE57338, whereas the single-cell RNA sequencing data were derived from a separate independent dataset. Given the discrepancies in technical principles and data sources between the two approaches, quantitative direct comparisons of their immune infiltration results are inappropriate. Nevertheless, single-cell sequencing data offer more precise cell-level evidence regarding alterations in immune cell populations.

Combining bioinformatics analysis with clinical sample validation, this study identified two Kbhb modification-related biomarkers (HMOX2 and KPNA2) in HF and delineated their roles in the proteasome, oxidative phosphorylation, and amino acid metabolism pathways via functional enrichment analysis. Additionally, immune infiltration and single-cell analysis demonstrated specific expression patterns of T cell subpopulations in HF, underscoring the importance of immune-metabolic interactions in the disease. Furthermore, molecular docking results imply that Kbhb may influence epigenetic regulation by affecting nuclear transport processes. While these findings offer valuable biological insights, certain limitations exist: primarily, the analysis relied on publicly available databases, and sample heterogeneity could impact the robustness of the conclusions; second, the number of clinical samples included in this study is limited.GO and KEGG enrichment analyses as well as PPI network analysis only provide a descriptive overview of biological pathways and protein–protein interaction networks potentially involved by candidate genes, and cannot directly infer the specific regulatory mechanisms through which Kbhb modification acts on these genes. Future investigations will need to adopt multiple approaches, including ChIP-seq, mass spectrometry-based identification of Kbhb modification sites, and gene-editing functional assays, to verify whether Kbhb directly modifies these candidate genes and regulates their functions in heart failure. In addition, the CIBERSORT algorithm was utilized in this study to analyze immune infiltration in myocardial tissue. However, this algorithm was originally developed based on blood and tumor samples, while cardiac tissue is predominantly composed of non-immune cells including cardiomyocytes, fibroblasts and endothelial cells. Therefore, the deconvolution results derived from myocardial samples possess limited reliability and should only be regarded as exploratory analyses [61,62]. Single-cell RNA sequencing or immunohistochemistry will be required in future research for further validation. Future research should incorporate animal models and myocardial tissue validation to explore the molecular mechanisms of key molecules, which could enhance their clinical translational potential.

## Supporting information

S1 Fig The scRNA-seq data processing. (**A-B**) Before and after quality control (QC) of GSE183852; (**C**) Visualization of the top 2000 highly variable genes (HVGs) in GSE183852; (**D-E**) The principal component analysis (PCA) of GSE183852.
(TIF)

S2 Fig Re-clustering analysis of T cells. (**A-B**) PCA of T cells in GSE183852; (**C**) UMAP plot of 9 clusters of T cells; (**D**) Expression of marker genes in different annotated subsets of T cells.
(TIF)

S1 Table Summary Table of KRGs. (CSV)

S2 Table Summary Table of GO KEGG analysis. (XLSX)

S3 Table Summary Table of KEGG analysis. (XLSX)

S4 Table Summary Table of drug prediction. (XLSX)

S5 Table ELISA Experimental Validation Data. (ZIP)
